# Unveiling Hyperinflammation in Severe Dengue With Coinfection Among Children: A Case Series

**DOI:** 10.7759/cureus.79812

**Published:** 2025-02-28

**Authors:** Adyasha Mishra, Bibekananda Mukherjee, Ratan Kumar, Kumar Diwakar, Preeti Srivastava

**Affiliations:** 1 Department of Pediatrics, Tata Main Hospital, Jamshedpur, IND; 2 Department of Pediatrics, Manipal Tata Medical College, Manipal Academy of Higher Education (MAHE), Jamshedpur, IND

**Keywords:** coinfection, hyperinflammation, ivig, organ dysfunction, severe dengue

## Abstract

Dengue illness follows three phases: febrile, critical, and convalescent. Coinfection and hyperinflammatory response are common causes of the persistence of fever beyond the febrile phase of dengue. However, finding both these causes together in severe dengue cases is rarely reported in the literature.

We present three cases of severe dengue who presented with persistent fever. The first case is of a 14-year-old girl who presented with dengue shock and culture-proven *Acinetobacter *sepsis. Later she was diagnosed with a hyperinflammatory response to infection and improved after intravenous immunoglobulin (IVIG) administration. The second case is of a 13-year-old boy who presented with severe dengue and culture-proven *Burkholderia *sepsis. He also had persistent fever despite appropriate treatment of infection which later improved after IVIG administration for hyperinflammation. The third case is of a 10-year-old girl who presented with severe dengue and encephalopathy. She was later diagnosed with coinfection with Japanese encephalitis and hyperinflammation.

These cases highlighted that coinfection and hyperinflammatory response can both together complicate dengue infection.

## Introduction

Dengue fever, a significant public health concern in tropical regions, can lead to complications, like hyperinflammatory response, particularly in children. Despite growing recognition, dengue-related hyperinflammation is still underdiagnosed and poses a challenge, as mortality is higher in severe dengue with hyperinflammation (39%) than in those without hyperinflammation (22%) [1]. Cytokine storm and cytokine release syndrome represent life-threatening inflammatory conditions characterized by immune cell hyperactivation and elevated cytokine levels, triggered by various infections, therapies, cancers, and autoimmune and genetic disorders [2].

The defining criterion for severe dengue includes either severe plasma leakage, severe bleeding, or severe organ dysfunction including impaired consciousness, myocardial dysfunction, or raised liver enzymes > 1000 U/L. Endothelial dysfunction, which causes increased vascular permeability and plasma leakage, is a hallmark of severe dengue. The progression to severe disease is determined by a combination of factors related to the patient and the pathogen [3]. The incidence of coinfection has been reported to be higher in severe dengue cases [4].

There is much literature suggesting that important causes for fever persisting beyond the febrile phase of dengue include coinfection and hyperinflammation. In this case series, we present three cases of hyperinflammation in children with severe dengue, associated with bacterial or viral coinfection. Simultaneously diagnosing both conditions in severe dengue cases is scarcely reported in the literature.

## Case presentation

Case 1

A 14-year-old previously healthy female child was admitted with a history of fever and 2-3 episodes of vomiting per day for four days and respiratory distress for one day. At admission, she was in compensated shock, which was treated with resuscitation fluid and other supportive treatment, including ceftriaxone as an empirical antibiotic. Initial investigations (Table 1) revealed leukopenia, thrombocytopenia, bilateral mild pleural effusion, dengue NS1 enzyme-linked immunosorbent assay (ELISA) positive, and scrub IgM ELISA negative. She was transfused with packed red blood cells (PRBC) in view of falling hematocrit and persistent shock. The blood culture report obtained after 72 hours of inoculation revealed growth of *Acinetobacter baumannii*; hence, the antibiotic was upgraded to meropenem as per sensitivity. Due to worsening pleural effusion, respiratory distress, and shock, she was intubated and started on inotrope support. By day 9 of illness, she continued to have a high spiking fever and inotrope requirement; hence, laboratory investigations for inflammatory markers were sent, which were raised. It was associated with pancytopenia and hepatomegaly; hence, a diagnosis of severe dengue with a hyperinflammatory response was made, and intravenous immunoglobulin (IVIG) was administered. In the next 48 hours, she became afebrile; inotrope and ventilator support was weaned off. Her platelet and total leukocyte count gradually improved, and she was discharged after 16 days of hospitalization following complete clinical recovery. She remained well till six months of follow-up.

**Table 1:** Laboratory reports of three cases Adm: laboratory value at admission; TLC: total leukocyte count; ANC: absolute neutrophil count; ALC: absolute lymphocyte count; ALT: alanine aminotransferase; AST: aspartate aminotransferase; PT: prothrombin time; INR: international normalized ratio; LDH: lactate dehydrogenase; ESR: erythrocyte sedimentation rate

Investigations	Case 1	Case 2	Case 3	Normal range
Hemoglobin (highest)	12.2	13	11.6	11.5-16.5 gm/dl
Hematocrit (highest)	38.7	40.9	38.8	35-50%
Hematocrit (lowest)	31.1	37.3	29.5	35-50%
RBC count (lowest)	2.9	4.1	3.0	4.1-5.5 million per cumm
Lowest platelet count	31,000	62,000	58,000	1,50,000-4,10,000 per cumm
TLC (Adm)	1,100	11,700	39,500	4,500-13,500 per cumm
ANC (Adm)	715	5031	5,925	2700-9450 per cumm
ALC (Adm)	341	5148	28045	1350-5400 per cumm
Serum sodium (Adm)	135	125	134	138-145 mEq/L
Serum potassium (Adm)	4.3	4.7	6.3	3.5-5.5 mEq/L
Serum calcium (Adm)	8.2	7.67	8.52	8.8-10.8 mg/dl
ALT (Adm)	133	1,178.2	645.7	0-45 U/L
AST (Adm)	317	3,448.4	1,699.3	0-35 U/L
PT (Adm)	15.6	20.6	15.1	10.3-16.5 seconds
INR (Adm)	1.17	1.57	1.13	0.8-1.2
Highest lactate	5.2	3.3	8.4	0.5-1.5 mmol/L
Urea (Adm)	23.4	58.3	71.5	15-36 mg/dl
Creatinine (Adm)	0.93	1.01	1.35	0.5-1 mg/dl
Total bilirubin (Adm)	0.51	1.65	4.75	0.2-1 mg/dl
Direct bilirubin (Adm)	0.29	0.83	3.10	0.1-0.5 mg/dl
LDH	516	1093	985.8	208-378 U/L
D-dimer	7.2	7.6	-	<0.5 mcg/ml
Serum ferritin	2215	7490	>15,000	11-306.8 ng/ml
Fibrinogen	341	-	-	200-400 mg/dl
Triglyceride	216.9	-	-	45-150 mg/dl
Interleukin 6	>1598	-	-	200-400 mg/dl
C-reactive protein (highest)	23.7	6.88	6.14	0.08-0.79 mg/dl
ESR (highest)	45	-	-	0.02-0.3 ng/ml
Procalcitonin (highest)	56.67	-	-	0.02-0.3 ng/ml

Case 2

A 13-year-and-18-month-old previously healthy male child with confirmed dengue fever (Dengue IgM ELISA positive) was admitted with fever for eight days and abdominal pain for three days. He had bilateral mild pleural effusion and tender hepatomegaly on examination. He was started on intravenous (IV) fluid as per the dengue protocol, along with other supportive treatments, including ceftriaxone as an empirical antibiotic. Liver enzymes (Table 1) were significantly raised (>1000 IU/ml), consistent with a diagnosis of severe dengue. By the 11th day of illness (day 3 of hospitalization), blood culture reported growth of *Burkholderia cepacia*, which was sensitive to third-generation cephalosporins; hence, ceftriaxone was continued. Despite this, high-grade fever persisted for the next 48 hours, prompting a work-up for hyperinflammation. Inflammatory markers were elevated, and serum ferritin was 7,490 ng/ml. He was diagnosed with a hyperinflammatory response and treated with IVIG, following which fever spikes resolved. After nine days of hospitalization and with improvement in clinical and laboratory parameters, he was discharged with no complications noted during the next six months follow-up in the outpatient department.

Case 3

A previously healthy 10-year-and-2-month-old female child was admitted with fever for seven days, loose stools and vomiting for two days, and abnormal tonic posturing of the whole body associated with up-rolling of the eyeballs and teeth clenching, lasting for 2-3 minutes, on the seventh day of illness. At admission, she had altered sensorium (Glasgow Coma Scale score = 9 (E2V2M5)), hypertonia, and extensor plantar reflex. Her vital parameters were within normal limits. She was started on anti-seizure medication (levetiracetam) and intravenous antibiotics, along with other supportive treatments.

Initial investigations were reported as dengue IgM ELISA positive, scrub IgM ELISA negative; thrombocytopenia; lymphocytic leukocytosis with raised C-reactive protein; deranged liver enzymes with direct hyperbilirubinemia; and deranged renal function, hyponatremia, and hyperkalemia (Table 1). Other infectious causes of acute hepatitis were ruled out. MRI of the brain with contrast showed meningeal enhancement. Cerebrospinal fluid (CSF) analysis showed 10 cells/mm³ (100% lymphocytes) and normal biochemical parameters; CSF ELISA for dengue was negative, but IgM ELISA for Japanese encephalitis came to be positive. Conservative management was continued, and her sensorium improved to normal by day 4 of hospitalization.

Despite treatment, she continued to have fever spikes beyond five days of hospital stay; hence, she was investigated for hyperinflammatory response. Finally, she was diagnosed with severe dengue and Japanese encephalitis with a hyperinflammatory response. She was advised IVIG on the 14th day of illness. But, because of personal reasons, her parents got her discharged against medical advice, and she was lost to follow-up.

## Discussion

In a retrospective observational study including 423 children with dengue fever, 83 children had persistent fever beyond that expected during the febrile phase of dengue, of whom 29 (34.9%) had confirmed sepsis (culture-proven), and 7 had probable sepsis (positive sepsis screen) [4]. Since it is clinically difficult to diagnose bacterial coinfection in severe dengue cases and mortality is high (14-44%) in cases with coinfection [5], antibiotics are often empirically prescribed while awaiting culture reports. The management is further challenging when fever persists despite appropriate treatment of coinfection.

Hyperinflammatory response to infection is suspected when fever persists beyond expected by the natural course of illness. Hyperinflammation is defined as persistent fever with one of the following criteria: marked hyperferritinemia, cytopenias, hepatomegaly and/or splenomegaly, or hemophagocytosis [6]. Like COVID-19, dengue can manifest as multi-organ impairment late in the disease course when viremia is declining and is likely to be driven by hyperinflammatory response [7].

The pathogenetic mechanism of coinfection in dengue cases can be vascular leakage, the disintegration of the mucocutaneous barrier, and changes in the immunological system [8]. The progression of disease and response to treatment deviates from the natural course in the presence of coinfection, which may lead to a delay in the diagnosis of hyperinflammatory response. The primary therapeutic options for infection-induced hyperinflammation include steroids, IVIG, plasma exchange, etoposide, and anakinra [6,9].

## Conclusions

Coinfection is common in severe dengue and is an important cause of fever persisting beyond the febrile phase. If fever persists despite appropriate management of coinfection, hyperinflammatory response to infection should be suspected. This case series supports emerging evidence that hyperinflammation is an important but often overlooked complication of severe dengue, highlighting the need for standardized diagnostic criteria and further research on optimal management strategies.
